# The Cold Welding Question

**Published:** 1859-10

**Authors:** 


### Editorial.

### THE COLD WELDING QUESTION.

At the New York meeting of the American Dental Convention, three years ago, the welding properties of gold were referred to, when some member suggested that there were but *two* welding metals, and that these were iron and platinum. We then remarked that this assumption was incorrect; for gold was pre-eminently the welding metal, as every worker in *pure* gold well knew. We had reference to the practice of gold beaters uniting fragments of leaves by pressure with a smooth-edged instrument. Prof. Austen remarked, (though in the report the statement is credited to Prof. Buckingham,) that if it were true that pressure would weld gold in a cold state, it was a new fact in chemistry. Prof. Buckingham, then, and afterward in the News Letter, maintained that the union of gold by pressure was not welding, but rather a species of riveting, as when two sheets of metal were laid together, and a punch was driven through both, at various points, they would adhere together. And, indeed, this theory leads to the inquiry as to the existence of welding properties in any metal. In uniting two pieces of hot iron, the particles of one may be driven into the pores of the other, and retained there by riveting.

Another theory was, that, as gold could only be thus united when freshly annealed, the union would not be permanent, but that the particles, losing their adhesiveness would separate. This was maintained, earnestly, by Mr. James Leslie, at a meeting of the Mississippi Valley Association. In advancing his theory he gave many interesting facts; and, finally, annealed three leaves of No. 6 foil, and united them by the pressure of his finger, suggesting, however, that they would, on exposure, lose their adhesiveness, and separate. We purchased these leaves, and exposed them "to all weathers," for ten months; and, throughout, they remained inseparable. Notwithstanding it contradicted his theory, no one was better pleased with the result than Mr. L.

Long before this, Dr. A. M. Leslie had maintained that gold foil was as adhesive as crystal gold; and we suppose, to this day, finds no good reason for a change of opinion.

But we set out to notice the progress of opinion, on this subject, in a few short years. All in the profession are now familiar with this property of gold; and it is almost, if not quite, unanimously regarded as *welding.* Prof. B., we believe, has dropped his rivet theory, and Prof. A. no longer regards it as a *new fact in chemistry,* but speaks of it in his late article on gold, as a well known and well established principle. Mr. L. is not concerned about the separation of welded gold, even though it be true that exposure interfers with, or destroys its adhesive properties; especially, when he remembers that though a reduction of temperature destroys the welding properties of iron, it does not separate two pieces already welded.

This subject affords us satisfactory evidence of the progressiveness of our profession; and the extensive use of adhesive foil, as recommended by Prof. Arthur, shows that practice keeps pace with theory. W.

